# Predictors of social distancing and hand washing among adults in five countries during COVID-19

**DOI:** 10.1371/journal.pone.0264820

**Published:** 2022-03-17

**Authors:** Kaston D. Anderson-Carpenter, Garrett S. Tacy

**Affiliations:** Department of Psychology, Michigan State University, East Lansing, Michigan, United States of America; Charite Universitatsmedizin Berlin, GERMANY

## Abstract

The purpose of this cross-sectional study is to examine disparities in hand washing and social distancing among 2,509 adults from the United States, Italy, Spain, the Kingdom of Saudi Arabia, and India. Respondents were recruited via Qualtrics’ participant pool and completed an online survey in the most common language spoken in each country. In hierarchical linear regression models, living in a rural area (β = -0.08, *p* = .001), older age (β = 0.07, *p* < .001), identifying as a woman (β = 0.07, *p* = .001), and greater educational attainment (β = 0.07, *p* = .017) were significantly associated with hand washing. Similar results were found regarding social distancing, in which living in a rural area (β = -0.10, *p* < .001), country of residence (β = 0.11, *p* < .001), older age (β = 0.17, *p* < .001), identifying as a woman (β = 0.11, p < .001), and greater educational attainment (β = 0.06, *p* = .019) were significant predictors. Results from the multivariable linear regression models demonstrate more nuanced findings with distinct and significant disparities across the five countries found with respect to hand washing and social distancing. Taken together, the results suggest multiple influencing factors that contribute to existing disparities regarding social distancing and hand washing among adults internationally. As such, more tailored public interventions are needed to promote preventive measures to mitigate existing COVID-related disparities.

## Introduction

To date, almost 200 million individuals have acquired COVID-19 globally; of those, more than 4 million have succumbed to the disease [1]. Although initial cases were confirmed at different times across countries, there exists striking differential impacts across continental regions [1]. At the time of this writing, data from the World Health Organization indicates that the Americas contain approximately 39.2% of worldwide COVID-19 infections, followed by Europe at 30.5% of the global prevalence [1]. Since the first documented case of COVID-19 in December 2019, international governments have implemented and enforced strict social distancing policies and encouraged frequent and proper hand washing to reduce transmission rates.

Despite the evidence for social distancing and hand washing on reducing viral transmissions, marginalized populations experience disparate rates COVID infection. For example, scholars have found that sexual minority (i.e., lesbian, gay, bisexual, pansexual, etc.) adults may have an elevated risk for infection compared to heterosexual peers [2]. Part of the COVID-related health disparities exist for sexual minorities due to intersectional identities [3, 4] related to minority stress [5, 6] and stigma, which place sexual minorities at a substantial disadvantage regarding COVID-19 acquisition and mortality.

COVID-related disparities have also been established between rural and urban populations, although the findings have been inconsistent. While Liu and [7] found that urban Chinese patients reported a higher prevalence of mental health concerns than their rural counterparts, scholars from the United States have found that rural adults endorsed greater COVID-related mental health concerns than their rural peers [8]. Moreover, rural states have performed COVID-19 testing at lower rates than their urban counterparts. Given the burgeoning literature on the psychosocial and behavioral implications of COVID-19, more systematic investigations are needed to understand and address such disparities and mitigate the pandemic’s negative effects across marginalized populations.

### Hand washing and social distancing as mitigation strategies

Both hand washing and social distancing have been identified as non-pharmaceutical public health interventions to combat the spread of COVID-19. International studies have long shown that proper and frequent hand washing is a simple, effective health behavior that can mitigate the risk of acquiring several viral and bacterial infections transmitted through skin-to-skin contact, respiration, or food [9, 10]. Godoy and colleagues [11] found that frequent hand washing and the use of alcohol-based hand sanitizers were protective factors against influenza acquisition requiring hospitalization, and other scholars noted the utility of frequent hand washing with soap and water to protect against cholera in Nigeria [12]. Although prior research has demonstrated notable increases in perceived efficacy of hand washing to prevent communicable diseases [13], compliance with proper hand washing remains low [14–16]. As such, more investigations are needed to identify mechanisms that contribute to disparities of proper hand hygiene in local and cross-cultural contexts.

Like effective hand washing, social distancing (i.e., maintaining a distance of at least six feet, or two meters, from individuals with whom one does not live) has been shown to be effective against the spread against certain respiratory viral conditions. Recent studies in international contexts confirm that social distancing may be effective in mitigating COVID-19 risk [17–19]. In the Kingdom of Saudi Arabia, for example, researchers found that taking a multi-faceted and systemic approach to facilitating social distancing measures may potentially mitigate the spread of COVID-19, especially in communities and cultures that highly value interconnectedness as social norms [17]. To be sure, successful implementation of widespread social-distancing measures require coordinated, multicomponent efforts across multiple ecological levels and community sectors. Nevertheless, the extant literature provides consensus on the potentially mitigative effects of proper social distancing on attenuating the spread of COVID-19.

### Sociodemographic determinants of social distancing and hand washing

To date, several studies have examined sociodemographic determinants of social distancing and hand washing in the COVID-19 context. For instance, older age is significantly associated with COVID-related preventive measures such as social distancing and hand washing [20–22]. Although age effects are typically measured in years, one study [20] found that the Baby Boomer generation (aged 56–74 years) endorsed greater social distancing than Millennials (aged 24–39 years). This approach adds a nuanced understanding of the relationship between age and COVID-related preventive measures, suggesting a potential generational effect. More broadly, the extant literature suggests that across international samples, age is a consistent predictor of COVID-related protective measures.

Similarly, investigations have demonstrated educational attainment and employment status impact social distancing and hand washing behaviors. Lüdecke and von dem Knesebeck [21] found that in a German sample, individuals with lower educational attainment were less likely to engage in social distancing or engage in appropriate hand washing behavior—although the observed effect sizes were small to negligible. Those findings were consistent with other work [23], which found that lower educational attainment and employment in agricultural and domestic labor (compared to business sector) predicted lower levels of social distancing and hand washing among Iranian adults.

Although research is consistent regarding the predictive effects of age, education, and employment on social distancing and hand washing, the same cannot be said for gender and urbanicity. Suen and colleagues [10], for example, found that women were more likely to perform proper hand hygiene techniques compared to men. Their findings were echoed in research among Bangladeshi men who have been found to have significantly greater odds of experiencing challenges with practicing COVID-related preventive behaviors [23]. However, in Saudi Arabian samples, men were more likely to wash their hands with soap and water than women [24]. Together, these studies point to inconsistent gender effects on hand washing behavior related to COVID-19 prevention.

Numerous scholars have highlighted the urban-rural disparities in COVID-19 morbidity and mortality [7, 8, 25]; however, limited work has examined urbanicity in relation to social distancing and hand washing. The extant literature suggests, similar to gender effects, that the evidence for the effect of urbanicity remains inconclusive. On the one hand, rural residents have shown to endorse lower levels of social distancing and hand washing [23], even though they present with greater knowledge of COVID-related prevention strategies [26]. On the other hand, urban dwellers have endorsed greater intent to practice social distancing and hand washing relative to their rural-dwelling counterparts [26]. Still, urban dwellers living in poverty may experience unique challenges in practicing social distancing and hand washing [27], suggesting that the syndemic nature of urbanicity and socioeconomic status may play a more substantial role in COVID-19 prevention than previously conceived. Taken together, the role of specific social determinants of health on social distancing and hand washing remains largely inconclusive. As such, the implementation of broader ecological approaches to mitigate COVID-19 risk is limited.

#### Rationale and hypotheses

Despite the utility of hand washing and social distancing measures in reducing the transmission of COVID-19, no study to date has examined existing disparities of hand washing or social distancing in an international context. Furthermore, the literature remains limited in examining the differential risks of subpopulations within and across multiple countries. To that end, we examine associations with hand washing and social distancing across five countries affected by the COVID pandemic. Based on prior literature [2, 7, 8], we hypothesize the following: (a) older age will be positively associated with greater hand washing and social distancing; (b) living in a non-urban setting (i.e., suburban or rural/village) will be associated with lower levels of hand washing and social distancing compared to living in an urban setting; and (c) identifying as a sexual minority (i.e., lesbian, gay, bisexual, and pansexual) will be associated with lower levels of hand washing and social distancing compared to identifying as heterosexual.

## Materials and methods

The Michigan State University Human Research Protection Board approved the study as exempt from IRB review (STUDY00004591). Each participant received a downloadable PDF version of the informed consent form written in their language. They provided an electronic response to indicate their informed consent to participate in the study. Participants for this study were recruited through Qualtrics, which has a large, representative pool of potential participants representing most of the countries in the world. Individuals were eligible for study inclusion if they were: (a) aged 18 and older; had Internet access; (b) not institutionalized or residing in a nursing facility; and (c) resided in the United States, Italy, Spain, the Kingdom of Saudi Arabia, or India during the COVID pandemic in the summer months of 2020. These five countries were selected for inclusion because they reported the highest prevalence rates of COVID-19 infections in their respective geographical regions as of May19, 2020 [28]. participants received an invitation link from Qualtrics (QualtricsXM, Provo, UT, 2020) and completed structured questionnaires in a survey from July-August 2020, which represented the peak incidence and prevalence in many countries.

The 20-minute survey was administered through Qualtrics, and all questionnaires within the survey were anonymous to ensure confidentiality and reliability of data. We used the Power and Sample Size 2020 software (PASS 2020) to calculate the estimated sample size. Using the conditional power calculation method, a sample size of 411 per country achieves 80% power to detect an R-squared of 0.02 using an F-test with a significance level of 0.05. Accounting for an approximate 20% attrition rate, the attrition-inflated sample size was 500 participants per country. The final analysis comprised individuals who identified as cisgender men or cisgender women (i.e., those who identify with the gender or sex assigned at birth) due to small subsamples of adults who disclosed non-cisgender identities.

### Instruments and measures

Social distancing was defined as engaging in socially distancing at least 6 feet (2 meters) from others, and adequate hand washing was defined as washing hands with soap and water for at least 20 seconds each time. Social distancing was measured using a COVID Protective Measures Scale developed by the author. The scale comprised four items (i.e., “I avoid large groups,” “I stay at least 2 meters (6 feet) away from others,” “I do not hug people or shake people’s hands,” “I wash my hands with soap and water for 20 seconds or more”) and was measured using Likert scaling (i.e., 1 = “strongly disagree” and 5 = “strongly agree”). Likewise, hand washing was measured on a Likert scale (1 = “strongly disagree” and 5 = “strongly agree”) using the item “I wash my hands with soap and water for 20 seconds or more.” Overall, the four items showed good internal consistency in the sample (Cronbach’s α = .83), and social distancing questions showed similar internal consistency (Cronbach’s α = .82). All items were translated and back-translated into Italian, Spanish, Arabic, and Hindi for participants residing in Italy, Spain, the Kingdom of Saudi Arabia, and India, respectively. The English version of the scale, along with the translations, are provided as S1 File.

Sexual orientation was a categorical variable measured with the item "Which of the following best describes your sexual orientation?" Response options included heterosexual, lesbian, gay, bisexual, and pansexual or other. Participants self-reported their gender identity as one of the following categories: cisgender man, cisgender woman, transgender man, transgender woman, nonbinary or gender fluid, and other gender identity. They were also given the option to select “I don’t know,” to fill in their gender identity, or decline to answer. Because only 27 participants identified as transgender, nonbinary, genderfluid, other gender identity, or unknown gender identity, we did not include those individuals in the final analysis due to inadequate sample size. Other categorical predictor variables included education, employment status; and age in years was entered as a continuous variable. Locality was a categorical variable measured using the item "What type of community best describes where you live?" Response options included: (a) “I live in a large (or major) city,” (b) “I live near a large (or major) city,” (c) “I live in a rural town or village,” and (d) “I decline to answer.”

### Data analysis

The full data set is available through the Open Science Foundation (https://osf.io/gdy3z/). Data were analyzed using Stata/SE version 15.1 (StataCorp, College Station, TX). Descriptive statistics were used to show sociodemographic characteristics of respondents overall and stratified by country. Chi-square tests were used to examine differences by country, and one-way ANOVA was used to identify whether significant age differences existed between countries. Statistically significant variables (i.e., *p* < .05) were screened and included in the multivariable linear regression analysis. Strengths of associations were assessed using both standardized and unstandardized estimates as well as standard errors, which are presented in the Results.

### Ethical considerations

The Michigan State University Institutional Review Board determined the study was exempt from review. All approvals and permits to conduct human subjects research in each participating country were obtained through Qualtrics as part of their process for administering research surveys to international participants. Prior to agreeing to participate, all respondents were provided with detailed informed consent forms regarding the purpose of the study, their responsibilities, risks and benefits of participating, and the privacy and protection of their data. All procedures for this study complied with university, local, state, federal, and international regulations on research with human participants. Each participant had the option of downloading a PDF version of the detailed informed consent form in their language for their records.

## Results and discussion

Table 1 displays descriptive statistics of demographic data for the overall sample and stratified by country. The mean age was 37.1 years (*SD* = 13.03 years), with a statistically significant difference between countries (*p* < .001). There was a roughly equal distribution of men (50.4%) and women (49.6%); however, the distributions by country were significantly different within and across countries (*p* < .001). In the United States, Italy, and Spain, women represented 65.0%, 62.8%, and 57.8% of the sample respectively. In the Saudi Arabian and Indian subsamples, however, women represented 31.3% and 31.2% of the countries’ respondents, respectively.

**Table 1:** Descriptive statistics of sociodemographic characteristics stratified by country (*N* = 2,482).

Sociodemographic characteristic	United States *n* (%)	Italy *n* (%)	Spain *n* (%)	Saudi Arabia *n* (%)	India *n* (%)	Total *N* (%)	Sig.
Age (*M*, SD)	36.5 (15.91)	40.5 (12.19)	35.6 (11.18)	40.0 (13.07)	32.8 (10.56)	37.1 (13.03)	<.001
Gender							<.001
Men	174 (35.0)	184 (37.2)	209 (42.2)	340 (68.7)	344 (68.8)	1,251 (50.4)	
Women	323 (65.0)	311 (62.8)	286 (57.8)	155 (31.3)	156 (31.2)	1,231 (49.6)	
Sexual orientation							<.001
Heterosexual	411 (83.5)	458 (94.6)	427 (87.9)	382 (84.0)	328 (73.1)	2,006 (84.8)	
Lesbian or gay	18 (3.7)	6 (1.24)	17 (3.5)	27 (5.9)	17 (3.8)	85 (3.6)	
Bisexual	45 (9.2)	13 (2.7)	32 (6.6)	12 (2.6)	46 (10.2)	148 (6.3)	
Pansexual or other	18 (3.7)	7 (1.5)	10 (2.1)	34 (7.5)	58 (12.9)	127 (5.4)	
Education							<.001
High school or less	167 (34.0)	216 (43.7)	122 (24.8)	78 (16.0)	22 (4.4)	605 (24.5)	
Some college	130 (26.5)	64 (13.0)	121 (24.6)	53 (10.8)	24 (4.8)	392 (15.9)	
College graduate	97 (19.8)	77 (15.6)	151 (30.7)	254 (52.0)	202 (40.5)	781 (31.7)	
Postgraduate	97 (19.8)	137 (27.7)	98 (19.9)	104 (21.3)	251 (50.3)	687 (27.9)	
Employment							<.001
Full-time	189 (38.9)	268 (54.8)	278 (58.0)	322 (66.3)	333 (66.7)	1,390 (57.0)	
Part-time	111 (22.8)	89 (18.2)	74 (15.5)	70 (14.4)	93 (18.6)	437 (17.9)	
Unemployed	118 (24.3)	70 (14.3)	88 (18.4)	51 (10.5)	59 (11.8)	386 (15.8)	
Retired	42 (8.6)	23 (4.7)	12 (2.5)	29 (6.0)	4 (0.8)	110 (4.5)	
Other	26 (5.4)	39 (8.0)	27 (5.6)	14 (2.9)	10 (2.0)	116 (4.8)	
Urbanicity							<.001
Urban	194 (39.5)	191 (38.7)	277 (56.3)	408 (83.1)	331 (66.3)	1,401 (56.8)	
Suburban	191 (38.9)	115 (23.3)	140 (28.5)	54 (11.0)	81 (16.2)	581 (23.6)	
Rural	106 (21.6)	187 (37.9)	75 (15.2)	29 (5.9)	87 (17.4)	484 (19.6)	

*Note*. Percentages may not add to 100% due to rounding.

The sample comprised an overwhelming majority of heterosexual adults (84.8%), with an additional 6.3% reporting being bisexual. Approximately 5.4% of respondents identified as pansexual or another sexual orientation not listed in the response options (e.g., asexual), with only 3.6% of the overall sample identifying as lesbian or gay. Significant differences in sexual orientation distributions were found between countries (*p* < .001). Although heterosexual respondents remained overrepresented in the sample, nuances were especially notable among sexual minority adults. For example, the greatest percentage of adults identifying as pansexual or other sexual orientation was in India (12.9%), yet the lowest concentration was in Italy (1.5%). Similarly, 10.2% of Indian respondents identified as bisexual, yet only 2.6% of Saudi Arabian respondents identified such. Apart from Saudi Arabia, each country’s respondents had the lowest percentage of lesbian or gay respondents.

A majority of the respondents (59.6%) had earned either a bachelor’s (31.7%) or postgraduate (27.9%) degree, and 24.5% earned a high school education or less. Analyses within and across countries revealed statistically significant differences in educational attainment (*p* < .001). The greatest concentration of respondents reporting a high school education or less were in the United States (34.0%) and Italy (43.7%), with the lowest concentrations reflected in the Saudi Arabian (16.0%) and Indian (4.4%) subsamples. It is also worth noting that in the Indian subsample, approximately 90.8% of the respondents attained either a bachelor’s (40.5%) or postgraduate (50.3%) degree.

A majority the respondents (57.0%) were full-time workers, with an additional 17.9% endorsing part-time employment status. Approximately 15.8% were unemployed at the time of the survey administration. Within and across countries, there were significant differences in employment status (*p* < .001). Full-time status was the most frequently reported in all five countries. However, part-time status was the second most reported employment status in the Italy (18.2%), Saudi Arabia (14.4%), and India (18.6%). Furthermore, unemployment was the second most frequently reported status in the United States (24.3%) and Spain (18.4%). We also found significant differences in the distribution of urbanicity, such that 56.8% of respondents lived in urban areas, 23.6% lived in suburban dwellings, and 19.6% lived in rural areas. Stratified by country, suburban dwelling was the second most reported area for respondents in the United States (38.9%), Spain (28.5%), and Saudi Arabia (11.0%). On the other hand, rural living was more prominent in Italy (37.9%) and India (17.4%) compared to suburban living.

### Social distancing

Table 2 shows the unstandardized and standardized effects of sociodemographic factors on practicing COVID-related social distancing in the overall sample and stratified by country. In the overall sample, age was significantly associated with social distancing (*b* = 0.03, β = 0.16, *p* < .001) with a small effect size. Country-stratified analyses showed similar effects of age in India (*b* = 0.04, β = 0.15, *p* = .002), Saudi Arabia (*b* = 0.04, β = 0.22, *p* < .001), Spain (*b* = 0.07, β = -0.28, *p* < .001), and Italy (*b* = 0.03, β = 0.13, *p* = .023). Women were also more likely than men to socially distance (*b* = -1.04, β = -0.16, *p* < .001), with greater effect sizes found in Italy (*b* = -1.04, β = -0.16, *p* < .001) and Spain (*b* = -1.04, β = -0.16, *p* < .001). No significant effects of gender on social distancing were found in the United States, Saudi Arabia, or India.

**Table 2:** Unstandardized and standardized multivariable linear regression weights for social distancing, stratified by country.

Predictor	Total		United States		Italy		Spain		Saudi Arabia		India	
	B (SE)	β	B (SE)	β	B (SE)	β	B (SE)	β	B (SE)	β	B (SE)	β
Age	0.03*** **(0.01)**	**0.16**	0.02 (.01)	0.10	0.03* **(0.01)**	**0.13**	0.07*** **(0.01)**	**0.28**	0.04*** **(0.02)**	**0.22**	0.04** **(0.01)**	**0.15**
Gender												
Men	Ref.	Ref.	Ref.	Ref.	Ref.	Ref.	Ref.	Ref.	Ref.	Ref.	Ref.	Ref.
Women	0.43*** **(0.12)**	**0.08**	0.38 (0.29)	0.06	0.71** **(0.25)**	**0.14**	0.80** **(0.25)**	**0.15**	0.40 (0.25)	0.07	0.46 (0.26)	0.08
Sexual orientation												
Heterosexual	Ref.	Ref.	Ref.	Ref.	Ref.	Ref.	Ref.	Ref.	Ref.	Ref.	Ref.	Ref.
Lesbian/gay	-0.75* **(0.38)**	**-0.05**	0.31 (0.82)	0.02	-2.23 (1.40)	-0.10	-0.17 (0.69)	-0.01	-1.16 (0.73)	-0.11	-1.30 (0.72)	-0.10
Bisexual	-0.25 (0.25)	-0.02	-0.43 (0.46)	-0.04	0.10 (0.74)	0.01	-0.33 (0.59)	-0.03	-1.25 (0.98)	-0.08	-0.37 (0.43)	-0.04
Pansexual/other	-0.06 (0.25)	-0.01	-0.48 (0.75)	-0.03	-1.09 (0.89)	-0.05	1.53 (0.96)	0.07	-0.19 (0.05)	-0.02	-0.65 (0.38)	-0.08
Education												
Postgraduate	Ref.	Ref.	Ref.	Ref.	Ref.	Ref.	Ref.	Ref.	Ref.	Ref.	Ref.	Ref.
College graduate	-0.01 (0.14)	-0.02	0.59 (0.43)	0.08	-0.68 (0.40)	-0.10	0.004 (0.35)	0.001	-0.62* **(0.23)**	**-0.12**	0.13 (0.24)	0.02
Some college	-0.38* **(0.18)**	**-0.05**	0.38 (0.43)	0.06	-0.78 (0.42)	-0.11	-0.30 (0.37)	-0.05	-0.73 (0.44)	-0.09	0.28 (0.75)	0.02
HS or less	-0.54** **(0.17)**	**-0.09**	0.18 (0.44)	0.03	-0.43 (0.30)	-0.09	0.40 (0.35)	0.07	-1.10** **(0.50)**	**-0.15**	-1.26 (1.08)	-0.09
Employment status												
Full-time	Ref.	Ref.	Ref.	Ref.	Ref.	Ref.	Ref.	Ref.	Ref.	Ref.	Ref.	Ref.
Part-time	-0.24 (0.16)	-0.03	-0.76* **(0.37)**	**-0.11**	0.17 (0.33)	0.03	-0.34 (0.36)	-0.05	0.09 (0.37)	0.01	-0.37 (0.35)	-0.05
Unemployed	0.06 (0.17)	0.01	-0.43 (0.38)	-0.07	-0.09 (0.33)	-0.01	-0.16 (0.33)	-0.02	0.02 (0.40)	0.002	0.34 (0.42)	0.40
Retired	-0.23 (0.30)	-0.02	0.33 (0.56)	0.03	0.66 (0.56)	0.05	-2.74** **(1.00)**	**-0.17**	-1.25 (0.70)	-0.12	0.58 (0.47)	0.02
Other	0.13 (0.25)	0.01	-0.28 (0.52)	-0.02	0.62 (0.49)	0.07	-0.26 (0.47)	-0.02	0.93 (0.56)	-0.05	-0.45 (0.95)	-0.02
Urbanicity												
Urban	Ref.	Ref.	Ref.	Ref.	Ref.	Ref.	Ref.	Ref.	Ref.	Ref.	Ref.	Ref.
Suburban	-0.38** **(0.14)**	**-0.01**	0.07 (0.30)	0.01	-0.05 (0.26)	-0.01	-0.40 (0.27)	-0.07	0.12 (0.46)	0.01	-0.84* **(0.40)**	**-0.11**
Rural	-0.72*** **(0.16)**	**-0.11**	0.23 (0.36)	0.03	0.39 (0.27)	-0.08	-0.42 (0.33)	-0.06	-1.45* **(0.58)**	**-0.13**	-1.03* **(0.44)**	**-0.14**

Note.

*p < .05;

**p < .01;

***p < .001.

HS = High School. Ref. = reference category for the categorical variable.

Differences by sexual orientation were also found but only in the overall sample. Specifically, lesbian and gay participants endorsed less social distancing compared to their heterosexual peers (*b* = -0.75, β = -0.05, *p* < .001). No significant differences were found in the overall sample with respect to employment status; in Saudi Arabia, however, both college graduates (*b* = -0.62, β = -0.12, *p* = .029) and those with a high school education or less (*b* = -1.10, β = -0.15, *p* = .009) endorsed less social distancing compared to postgraduates. Compared to adults who completed postgraduate school, those who completed some college (*b* = -0.38, β = -0.05, *p* = .003) and high school or less (*b* = -0.45, β = -0.07, *p* = .003) were less likely to socially distance from their peers.

Although we found no significant effects of employment status in the overall sample, stratified analyses showed that retired adults in Spain were significantly less likely to engage in social distancing compared to those employed full-time (*b* = -2.74, β = -0.17, *p* = .006). In the United States, part-time employees were also significantly less likely to engage in social distancing compared to full-time workers (*b* = -0.76, β = -0.11, *p* = .041), although the effects were smaller than those for retired Spanish respondents. We also found that both suburban (*b* = -0.38, β = -0.01, *p* = .005) and rural (*b* = -0.72, β = -0.11, *p* < .001) respondents were significantly less likely to practice social distancing compared to their urban counterparts, with stronger effects shown for rural respondents. Similar effects were shown among Indian respondents living in suburban (*b* = -0.84, β = -11, *p* = .034) and rural (*b* = -1.45, β = -0.14, *p* = .020) settings compared to their urban-dwelling peers. Furthermore, rural residents in Saudi Arabia were significantly less likely to practice social distancing than their counterparts in urban settings (*b* = -1.45, β = -0.13, *p* = .013).

To assess the specific effects of sexual orientation and locality on social distancing, we conducted a three-block hierarchical linear regression model (Table 3). In the first model (R^2^ = 0.002, *p* = .023), sexual minority status was associated with less social distancing (*b* = -0.16, β = -0.07, *p* = .023). The addition of urbanicity and country of residence in Model 2 added 2.2% (*p* < .001) to the variance in Model 1, bringing the total variance explained to 2.5% (*p* < .001). In Model 2, sexual minority status remained significantly associated with less social distancing (*b* = -0.20, β = -0.06, *p* = .004). Moreover, living in nonurban settings was negatively associated with social distancing (*b* = -0.30, β = -0.09, *p* < .001). However, participants who lived outside the United States endorsed more social distancing overall compared to United States residents (*b* = 0.18, β = 0.10, *p* < .001).

**Table 3:** Hierarchical linear regression of social distancing on sociodemographic predictors with standardized and unstandardized coefficients.

Predictor	Model 1			Model 2			Model 3		
	B	SE	β	B	SE	β	B	SE	β
Sexual orientation (ref: heterosexual)	**-0.16** *	**0.07**	**-0.05**	**-0.20** **	**0.07**	**-0.06**	-0.13	0.07	-0.04
Urbanicity (ref: urban)				**-0.30** ***	**0.07**	**-0.09**	**-0.31** ***	**0.08**	**-0.10**
Country (ref: United States)				**0.18** ***	**0.04**	**0.10**	**0.21** ***	**0.05**	**0.11**
Age							**0.03** ***	**0.04**	**0.17**
Gender (ref: men)							**0.56** ***	**0.12**	**0.11**
Education (ref: postgraduate)							**0.14** *	**0.06**	**0.06**
Employment (ref: full-time)							0.01	0.05	0.004
R^2^	**0.002** *			**0.025** ***			**0.059** ***		
ΔR^2^				**0.22** ***			**0.035** ***		

Note.

*p < .05;

**p < .01;

***p < .001.

“Ref.” refers to reference category for categorical variables.

The final model added 3.5% to the variance reported in Model 2 (*p* < .001), with a total of 5.9% of the variance explained by the full model (*p* < .001). In the full model, urbanicity (*b* = -0.31, β = 0.10, *p* < .001) and country of residence (*b* = 0.21, β = 0.11, *p* < .001) remained significant predictors of social distancing. Additionally, older adults (*b* = 0.03, β = 0.17, *p* < .001), women (*b* = 0.56, β = 0.11, *p* < .001), and those with greater educational attainment (*b* = 0.14, β = 0.06, *p* = .019) were more likely to engage in social distancing compared to younger adults, men, and those who completed high school or less, respectively. However, there were no significant effects of sexual orientation or employment status in the full model.

### Hand washing

Table 4 displays the unstandardized and standardized effects of social cofactors on practicing hand washing during COVID in the overall sample and stratified by country. Older age was significantly associated with greater hand washing in the overall sample (*b* = 0.01, β = 0.07, *p* = .006) and among Spanish respondents (*b* = 0.01, β = 0.004, *p* = .003). We also found that women endorsed more hand washing than men (*b* = 0.12, β = 0.07, *p* = .004) and in the Indian subsample (*b* = 0.19, β = 0.09, *p* = .047). Differences by sexual orientation were also found; lesbian and gay adults in the full sample were less likely to engage in hand washing relative to their heterosexual peers (*b* = -0.35, β = -0.07, *p* = .015). Among Italian respondents, adults who identified as bisexual reported greater hand washing compared to heterosexuals (*b* = 0.56, β = 0.10, *p* < .001), yet those who identified as and pansexual or other sexual orientation were less likely to practice adequate hand washing compared to heterosexuals (*b* = -1.02, β = -0.13, *p* < .001). Additionally, adults working part-time were less likely to engage in hand washing relative to adults working full-time (*b* = -0.11, β = -0.04, *p* = .047). Although no significant differences were found among respondents in India, Saudi Arabia, Spain, or Italy, part-time workers in the United States were significantly less likely to engage in proper hand washing compared to those working full-time (b = -0.33, β = -0.15, *p* = .011). Finally, suburban (*b* = -0.12, β = -0.06, *p* = .014) and rural (*b* = -0.13, β = -0.05, *p* = .022) respondents were significantly less likely to practice appropriate hand washing compared to their urban peers. Subgroup analyses showed that Indian suburban (b = -0.31, β = -0.11, *p* = .026) and rural residents (b = -0.44, β = -0.17, *p* = .009) were significantly less likely than urban dwellers to engage in hand washing. Among Saudi Arabian respondents, rural residents endorsed significantly less engagement in proper hand washing compared to their urban peers (b = -0.61, β = -0.14, *p* = .005).

**Table 4:** Unstandardized and standardized multivariable linear regression weights for hand washing behavior, stratified by country.

Predictor	Total		United States		Italy		Spain		Saudi Arabia		India	
	B (SE)	β	B (SE)	β	B (SE)	β	B (SE)	β	B (SE)	β	B (SE)	β
Age	0.01** **(0.002)**	**0.07**	-0.002 (0.004)	-0.03	0.001 (0.004)	0.01	0.01** **(0.004)**	**0.15**	0.01 (0.004)	0.12	0.01 (0.01)	0.09
Gender												
Men	Ref.	Ref.	Ref.	Ref.	Ref.	Ref.	Ref.	Ref.	Ref.	Ref.	Ref.	Ref.
Women	0.12** **(0.04)**	**0.07**	0.16 (0.10)	0.08	0.09 (0.09)	0.05	0.18 (0.09)	0.09	0.13 (0.10)	0.07	0.19* **(0.09)**	**0.09**
Sexual orientation												
Heterosexual	Ref.	Ref.	Ref.	Ref.	Ref.	Ref.	Ref.	Ref.	Ref.	Ref.	Ref.	Ref.
Lesbian or gay	-0.35* **(0.14)**	**-0.07**	-0.20 (0.27)	-0.04	-0.59 (0.65)	-0.07	-0.12 (0.22)	-0.02	-0.48 (0.28)	-0.12	-0.32 (0.35)	-0.06
Bisexual	-0.02 (0.08)	-0.05	-0.06 (0.14)	-0.02	0.56*** **(0.14)**	**0.10**	-0.08 (0.17)	-0.02	-0.76 (0.41)	-0.13	-0.08 (0.14)	-0.03
Pansexual or other	-0.05 (0.09)	-0.02	-0.45 (0.28)	-0.09	-1.02*** **(0.28)**	**-0.13**	0.64 (0.21)	0.08	-0.03 (0.14)	-0.01	-0.06 (0.14)	-0.02
Education												
Postgraduate	Ref.	Ref.	Ref.	Ref.	Ref.	Ref.	Ref.	Ref.	Ref.	Ref.	Ref.	Ref.
College graduate	0.04 (0.05)	0.02	0.18 (0.14)	0.08	-0.06 (0.12)	-0.02	0.14 (0.13)	0.07	-0.08 (0.10)	-0.04	-0.02 (0.09)	-0.01
Some college	-0.08 (0.07)	-0.03	0.09 (0.15)	0.04	-0.15 (0.14)	-0.06	-0.03 (0.14)	-0.04	0.06 (0.15)	0.02	0.17 (0.27)	0.04
High school or less	-0.14* **(0.06)**	**-0.06**	0.17 (0.16)	0.08	-0.16 (0.11)	-0.09	0.13 (0.15)	0.06	-0.43* **(0.18)**	**-0.16**	-0.64 (0.42)	-0.12
Employment												
Full-time	Ref.	Ref.	Ref.	Ref.	Ref.	Ref.	Ref.	Ref.	Ref.	Ref.	Ref.	Ref.
Part-time	-0.13* **(0.06)**	**-0.05**	-0.33* **(0.13)**	**-0.15**	0.09 (0.11)	0.04	-0.25 (0.13)	-0.10	-0.06 (0.13)	-0.02	-0.12 (0.13)	-0.05
Unemployed	0.02 (0.06)	-0.01	-0.22 (0.12)	-0.10	0.01 (0.12)	0.003	-0.03 (0.11)	-0.01	-0.10 (0.15)	-0.03	0.05 (0.17)	0.01
Retired	-0.10 (0.11)	-0.02	-0.15 (0.21)	-0.05	0.07 (0.24)	0.02	-0.38 (0.36)	-0.06	-0.23 (0.27)	-0.06	0.25 (0.29)	0.02
Other	-0.01 (0.10)	-0.001	-0.14 (0.20)	-0.03	-0.03 (0.18)	-0.01	0.06 (0.17)	0.02	-0.11 (0.22)	0.02	-0.06 (0.41)	-0.01
Urbanicity												
Urban	Ref.	Ref.	Ref.	Ref.	Ref.	Ref.	Ref.	Ref.	Ref.	Ref.	Ref.	Ref.
Suburban	-0.15** **(0.05)**	**-0.07**	0.07 (0.10)	0.04	-0.03 (0.10)	-0.01	-0.18 (0.10)	-0.09	-0.11 (0.16)	-0.04	-0.31* **(0.14)**	**-0.11**
Rural	-0.16** **(0.06)**	**-0.07**	0.12 (0.12)	0.05	0.03 (0.09)	0.02	-0.06 (0.12)	-0.02	-0.61** **(0.22)**	**-0.14**	-0.44** **(0.17)**	**-0.17**

Note.

*p < .05;

**p < .01;

***p < .001.

HS = High School. Ref. = reference category for the categorical variable.

Table 5 presents the results from a three-block hierarchical linear regression model regressing handwashing on sociodemographic predictors. In the first model (R^2^ = 0.002, *p* = .064), sexual orientation was not a significant predictor of hand washing behavior (*b* = -0.05, β = -0.04, *p* = .064). However, in the second model (R^2^ = .012, *p* < .001), sexual minority status (*b* = -0.05, β = -0.04, *p* = .045) and non-urban residence (*b* = -0.10, β = -0.08, *p* < .001) were negatively associated with hand washing as a protective behavior. Country of residence was not a significant predictor in the second model, regardless of participants’ sexual orientation or urbanicity.

**Table 5:** Hierarchical linear regression of hand washing on sociodemographic predictors with standardized and unstandardized coefficients.

Predictor	Model 1			Model 2			Model 3		
	B	SE	β	B	SE	β	B	SE	β
Sexual orientation (ref: heterosexual	-0.05	0.03	-0.04	**-0.05** *	**0.03**	**-0.04**	-0.04	0.03	-0.03
Urbanicity (ref: urban)				**-0.10** ***	**0.03**	**-0.08**	**-0.09** **	**0.03**	**-0.08**
Country (ref: United States)				0.03	0.01	0.04	0.02	0.02	0.04
Age							**0.01** **	**0.002**	**0.07**
Gender (ref: men)							**0.14** **	**0.04**	**0.07**
Education (ref: postgraduate)							**0.05** *	**0.02**	**0.06**
Employment (ref: full-time)							-0.01	0.19	-0.02
R^2^	0.002			**0.012** ***			**0.023** ***		
ΔR^2^				**0.01** ***			**0.011** ***		

Note.

*p < .05;

**p < .01;

***p < .001.

The final model explained 5.9% of the overall variance (*p* < .001) and represented a 3.5% increase in the variance explained in Model 2. In the final model, non-urban residence was associated with less hand washing (*b* = -0.09, β = -0.08, *p* = .001). However, older age (*b* = 0.01, β = 0.07, *p* < .001), identifying as a woman (*b* = 0.14, β = 0.07, *p* = .001), and greater educational attainment (*b* = 0.5, β = 0.06, *p* = .017) were all positively associated with hand washing in our sample. We did not find significant effects for country of residence, sexual minority status, or employment status on hand washing in the full model.

## Discussion

To our knowledge, this study is the first to systematically examine disparities in COVID-related social distancing and hand washing disparities in an international context. In the hierarchical models, older age was associated with greater adherence to social distancing and hand washing measures. These findings may be because older adults are at greater risk of presenting with pre-existing conditions that exacerbate the effects of COVID. Older adults remain a vulnerable population for COVID infection, and public health has targeted much of its messaging to the population. Given the disparity among younger adults in utilizing mitigation strategies, future research should systematically examine the mechanisms that drive such age-related disparities, particularly in international contexts.

### Sexual orientation and gender

In the hierarchical linear regression models, we did not find evidence for sexual minority adults experiencing disparately less social distancing or hand washing behavior compared to heterosexuals. However, analyses by country revealed that lesbian women and gay men endorsed greater levels of social distancing and hand washing than their heterosexual peers. Furthermore, Italian bisexual, pansexual, and other sexual minority adults reported significantly less hand washing compared to Italian heterosexuals. However, the effects for sexual minority adults were small to negligible. Thus, our hypothesis was partially supported.

Research suggests that community connectedness may suppress the deleterious effects of minority stress on health-related outcomes [29, 30]. However, the COVID-19 pandemic presents unique public health limitations on social gatherings that may run counter to the protective nature of close gatherings among sexual minority populations. Yet, sexual minority individuals in our sample may have utilized online communities to foster connectedness, support, and disseminating COVID-related mitigation strategies such as social distancing and hand washing. To this end, future investigations can investigate the comparative effects of in-person versus online connectedness to the LGBTQ+ community with respect to COVID conditions.

Overall, women reported greater social distancing and adherence to proper hand washing compared to men. Stratified analyses showed that Italian and Spanish women were significantly more likely to practice social distancing than their male peers, and Indian women were significantly more likely to engage in proper hand washing compared to Indian men. The observed effect of gender may be because in many cultures, women are often the family caregivers, and the COVID-19 pandemic may have increased the sense of cultural responsibilities for women to keep their families safe [31]—particularly women in India, Spain, and Italy. Additionally, the act of social distancing and hand washing may have been protective measures against psychological distress for women in our sample. In a recent systematic review of the literature [32], scholars have found that women experienced higher levels of anxiety, depression, and stress related to the COVID-19 pandemic. Thus, engaging in mitigation strategies may be a direct or indirect coping strategy for COVID-related psychological distress.

### Urbanicity

There was also an effect of urbanicity on social distancing and hand washing, but multivariable analyses by country showed significant effects only for respondents living in India and Saudi Arabia. Non-urban dwellers in these two countries may not have the ability or financial means to live in areas that are conducive to social distancing. Although awareness of social distancing and hand washing during the COVID-19 pandemic have been documented in Saudi Arabia [17, 24], other scholars have found disparities in implementing mitigation strategies in the Saudi population [33]. In India, social distancing and proper hand washing are not as prevalent as other mitigation strategies [34]. In rural areas in India, clean water is not as readily available to residents as in more urbanized areas [35, 36]; as such, environmental disparities may exacerbate existing disparities in hand hygiene.

Furthermore, rural residents may not have equitable access to reputable public health information compared to their urban-dwelling peers. It may also be the case that some respondents, particularly men, living in rural areas provide the majority of household income and may not be able to socially distance at work. Because the research in this area is new, additional investigations are needed to examine the mechanisms that drive disparities in COVID-related mitigation strategies among suburban and rural adults, especially in areas such as India and Saudi Arabia. Such research could further support public health efforts in promoting social distancing and proper hand washing among nonurban residents in these countries.

### Education and employment status

Results from the hierarchical regression models demonstrate that greater educational attainment was associated with greater social distancing and hand washing. Although the effects were negligible in the overall sample, stratified analyses revealed that slightly larger effect sizes were found among Saudi adults with a high school education or less. Moreover, employment was not a significant predictor of social distancing or hand washing in the hierarchical regression model. That said, part-time workers in the United States experienced significantly less social distancing and proper hand washing than adults working full-time. Although the literature on the specific contributions of educational attainment and employment status on social distancing and hand washing is limited, our findings correspond with studies demonstrating significant associations between social determinants of health, such as education and employment, and practicing mitigation strategies to minimize risk of respiratory-related communicable diseases [21, 26, 37–39]. Overall, our results, interpreted in context of the existing literature, suggest that the impact of education and employment on COVID-related social distancing and hand washing may be more nuanced than the literature may indicate. Furthermore, our findings warrant greater scientific investigations in the disparities experienced by residents of Saudi Arabia in an effort to reduce COVID infection and enhance their existing efforts to promote public health initiatives to address COVID-19.

### Limitations

Interpreting this study’s results comes with extreme caution due to its limitations. First, the cross-sectional nature of the study prohibits causal inference. Second, the self-report nature of the study increases the risk of both social desirability and recall biases. To address recall bias, future research should consider and integrate behavior-analytic technology, which has been shown to be effective at increasing COVID-related protective behaviors in other populations and settings [40–42]. Third, despite the study drawing from a large sample of adults across five countries, Internet-based recruiting may have limited efforts to recruit from geographic locations that lack high-speed Internet access. Given the stigma of being lesbian, gay, or bisexual that remains in many countries, participants in this study may have been reluctant to identify as a sexual minority and therefore indicated a heterosexual sexual orientation for the study. Moreover, due to international privacy concerns, we did not include variables such as household size, marital status, and other associated variables.

At the time of the study’s implementation, wearing face masks and other mitigation strategies were not universally required across all five countries in the study; therefore, we lack the data to draw inferences on such strategies. However, future research should investigate disparities in such mitigation strategies within and across marginalized populations. Finally, we did not analyze race/ethnicity in this international study because the categorization of race/ethnicity varies widely across countries, and many do not document race/ethnicity/nationality in the same ways as the United States. Therefore, it would have been impossible to accurately and completely document the nuanced racial/ethnic identities of the target populations.

## Conclusions

Limitations notwithstanding, this study demonstrates multiple nuanced disparities exist regarding social distancing and hand washing among adults globally. Although the inclusion criteria limited the number of countries included for investigation and limited the scope of generalizability, the international nature of the study reveal potential cross-cultural factors of hand washing and social distancing. The scientific literature strongly supports public health strategies to mitigate the impact of COVID-19. However, the results from this study suggest that blanket, one-size-fits-all approaches may result in disproportionate adherence, thus widening existing disparities. Thus, future investigations should further examine disparities in COVID-related protective behaviors in international contexts as well as refine existing approaches to be more sensitive and responsive to local community contexts. Relatedly, based on the findings of Mat Dawi and colleagues [43], future research and public health approaches should leverage the power of digital platforms from an ecological perspective. As vaccines have become more readily available, it remains critical to identify COVID-related disparities across subpopulations. By doing so, both scientists and practitioners can develop effective, culturally-tailored interventions to mitigate the harmful effects of COVID-19 and reduce the existing disparities in practicing social distancing and hand washing.

## Supporting information

S1 File COVID protective measures all languages. This file contains the COVID-19 Protective Measures scale in English, Spanish, Italian, Arabic, and Hindi.
(DOCX)
