# Supplementary material for: Predictors of social distancing and hand washing among adults in five countries during COVID-19
Source: PLoS One. 2022 Mar 17;17(3):e0264820. doi: 10.1371/journal.pone.0264820 (PMC8929564; doi:10.1371/journal.pone.0264820)

**COVID-19 Protective Measures (English)**

**Instructions:** Select the response that best reflects your social distancing activities

since the COVID-19 (coronavirus) pandemic affected your country. Try not to spend

too much time thinking about each statement but respond as quickly and honestly

as you can.

**
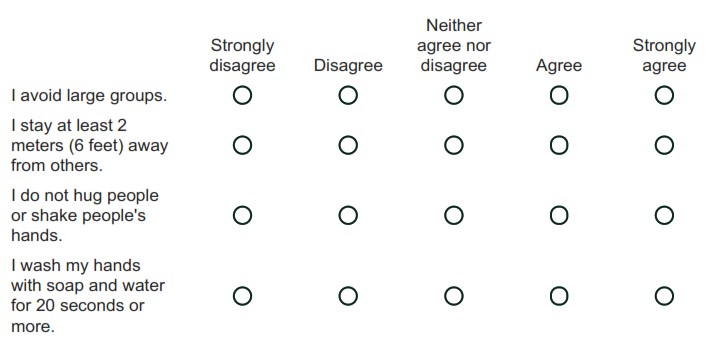
**

**COVID-19 Protective Measures (Spanish)**

**Instrucciones:** Seleccione la respuesta que mejor refleje sus actividades de distanciamiento social desde que la pandemia del COVID-19 (coronavirus) afectó a su país. Trate de no dedicar demasiado tiempo a reflexionar sobre cada afirmación y responda con la mayor rapidez y sinceridad posible.


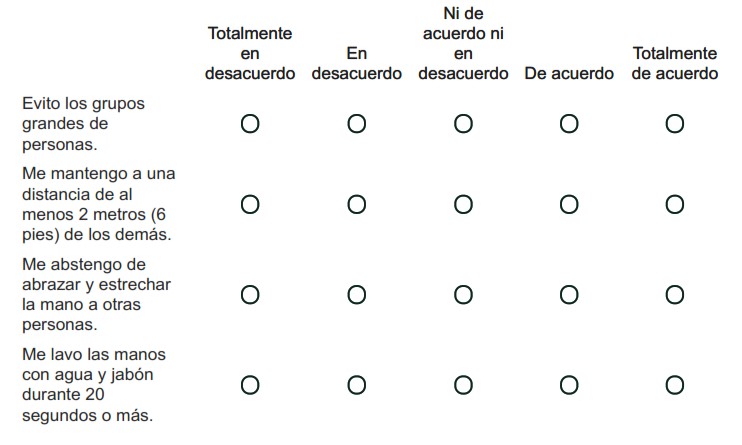


**COVID-19 Protective Measures (Italian)**

**Istruzioni:** Selezioni la risposta che descrive meglio le misure di distanziamento

sociale che ha adottato da quando la pandemia di COVID-19 (coronavirus) ha

colpito il Suo Paese. Cerchi di non soffermarsi a lungo su ogni frase, ma risponda

con la massima rapidità e sincerità.


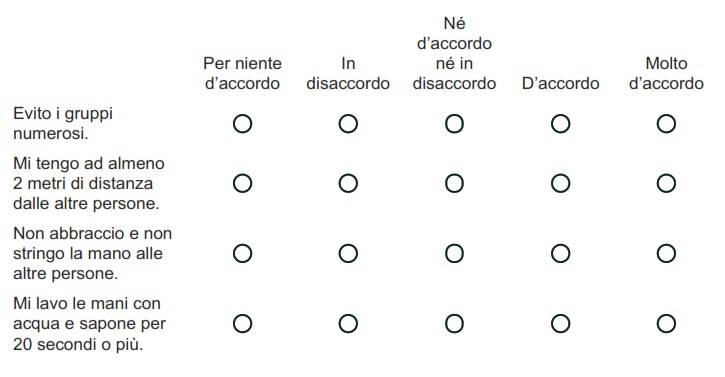


**COVID-19 Protective Measures (Hindi)**


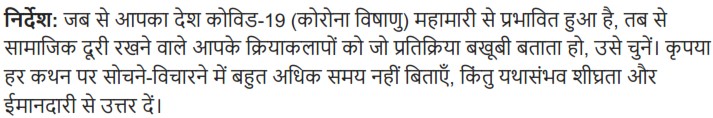


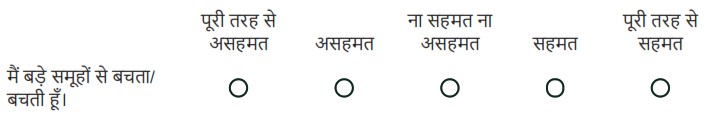


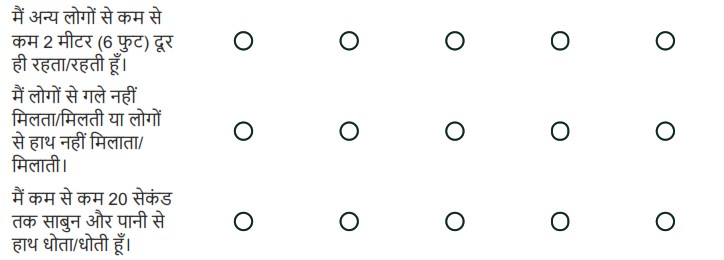


**COVID-19 Protective Measures (Arabic)**


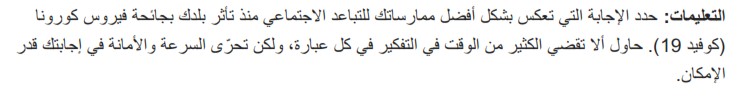


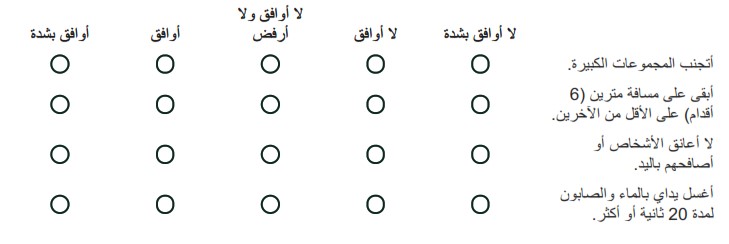

Supplement: S1 File — This file contains the COVID-19 Protective Measures scale in English, Spanish, Italian, Arabic, and Hindi. (DOCX) [file pone.0264820.s001.docx]
